# Transmission Electron Microscopy of XDR *Mycobacterium tuberculosis* Isolates Grown on High Dose of Ofloxacin

**DOI:** 10.3390/scipharm85010003

**Published:** 2017-01-24

**Authors:** Mohammad Arjomandzadegan, Maryam Sadrnia, Leonid Titov, Larissa Surkova, Hossein Sarmadian, Reza Ghasemikhah, Hossein Hosseiny

**Affiliations:** 1 Infectious Diseases Research Center (IDRC), Arak University of Medical Sciences, Arak 3848176941, Iran; arjomandzadegan@arakmu.ac.ir (M.A.); Dr.sarmadian@arakmu.ac.ir (H.S.); ghasemikhah@yahoo.com (R.G.); rhhosseiny@yahoo.com (H.H.); 2 Department of Biology, Payame Noor University, Tehran 79134-13915, Iran; 3 Research Institute for Epidemiology and Microbiology, Minsk 220114, Belarus; leonidtitov@tut.by; 4 Institute for Pulmonology and Phthisiology, Minsk 220114, Belarus; larissasurkova@tut.by

**Keywords:** *Mycobacterium tuberculosis*, transmission electron microscope, XDR, ofloxacin

## Abstract

The aim of the study was to investigate behavior of resistant *Mycobacterium tuberculosis* (MTB) isolates under a high dose of ofloxacin and its morphological changes. 19 extensively drug resistant (XDR) clinical isolates of MTB were grown on Löwenstein–Jensen medium containing progressively increasing concentrations of ofloxacin (2, 4, 8, 16, 32 mg/L). Ultra-structure analyses of resistant isolates grown on ofloxacin were conducted by transmission electron microscopy (TEM). Fixation was carried out by 4% glutaraldehyde in 0.1 M sodium cacodylate buffer on 300 mesh carbon formvar copper grid. The samples were negatively stained with uranium acetate suspension. All 19 XDR MTB isolates were grown and formed colonies successfully on 2, 4, 8 mg/L, seven isolates on 16 mg/L, and four isolates on 32 mg/L ofloxacin. Morphological changes and unusual forms were detected in 8, 16 and 32 mg/L ofloxacin at 43%, 76.5% and 81% of cells, respectively. Swollen form (protoplast like), ghost-like cell, degraded forms, and in a few cases, detached cytoplasm from cell wall were clearly detected in high drug concentrations in comparison to control. Changes in morphology were increased with increasing ofloxacin concentrations (*p* < 0.05). Some XDR isolates could be successfully grown on high doses of ofloxacin (32 mg/L), but with changes in morphology. It was concluded that several magnitudes of the drug doses could not prevent growth of drug resistant forms.

## 1. Introduction

Tuberculosis is a dangerous disease globally, with 9 million new cases each year [1]. About one third of the world’s population is infected with *Mycobacterium tuberculosis* (MTB) and 20 million are affected with tuberculosis [2]. Tuberculosis (TB) has greatly impacted the morbidity and mortality of humanity. MTB—the etiological agent responsible for the disease TB—remains the main cause of human mortality worldwide and could be resisted to a series of drugs through the history [3]. There are almost 2.7 million deaths caused by TB each year, and it has been estimated that approximately 2 billion people are harboring latent TB. This potential reservoir for active TB and the high mortality rate each year attributed to TB indicate that TB remains a serious public health threat [3].

Currently, TB is treated with four antibiotics. In resistant cases, treatment lasts from 1–2 years [4]. This is quite notable where ordinary bacterial infections are typically treated with antibiotics in only 10–14 days. Treatment of TB patients with anti-tuberculosis drugs is related to a number of factors. This bacillus grows slowly, so antibiotic therapy should be prolonged proportionately to effectively destroying all MTB cells in the host. Since antibiotics destroying active cells (replicating forms), the fact that MTB may reside in an inactive form or in a latent state in the host is a very complicating factor and requires prolonged treatments [5].

TB patients under prolonged antibiotic treatment regimens are notoriously weak, and it is common for patients to have incomplete treatment that results in drug-resistant MTB strains circulating in the human population. The frequency of these events is startling and has led to multiple drug-resistant MTB (MDR-TB, TB which does not respond to both isoniazid and rifampicin simultaneously), and extensively drug resistant TB (XDR-TB) with additional resistance to more anti-tuberculosis drugs. In many cases, MDR-TB and XDR-TB is impossible to successfully treat or is very costly [2,6].

There has been a renewed interest in the quinolones (a class of antimicrobial drugs) during the last decade, and new synthetic fluoroquinolones such as norfloxacin, ofloxacin, lomefloxacin, and sparfloxacin have been found to be very potent against MTB. Many of these compounds (particularly ofloxacin) can penetrate the cells and is active against intracellular MTB [7,8]. Ofloxacin is highly active against MTB, and is an important drug in tuberculosis treatment [3,7,9].

Ofloxacin was developed in 1982 as an analog of norfloxacin (the first fluoroquinolone antibiotic), and received the approval of the U.S. Food and Drug Administration (FDA) in 28 December 1990 [3,10,11].

Ofloxacin is a second-generation fluoroquinolone effective on DNA gyrase (type II topoisomerase) and topoisomerase IV, which is an enzyme necessary to separate replicated DNA, thereby inhibiting bacterial cell division [12,13].

Like other quinolone drugs, ofloxacin has been associated with a number of serious side effects, such as spontaneous ruptures of tendon and irreversible peripheral neuropathy, and in severe cases, may result in lifelong disabilities [14].

There are many ultrastructural studies on MTB. The mycobacterium cell wall skeleton is comprised of peptidoglycan, the plasma membrane is mostly comprised of phosphatidylinositol mannosides (PIMs), and the outer layer of the envelope is comprised of mycolic acids and lipoarabinomannan (LAM). This outer layer has many long-chain diols, such as phenolic glycolipids and mycosidic waxes [12,14].

The MTB cell wall is full of lipids, and it has been described as wax-like; some ultrastructural analyses attempted to describe the structure with chemical entities contained within the MTB cell wall [3,13]. The growth of MTB on culture medium containing a 2 mg/L concentration of ofloxacin means that the bacterium is resistant to ofloxacin.

The aim of the study was to investigate the behavior of ofloxacin-resistant MTB grown on very high doses of ofloxacin and any morphological changes during exposure to ofloxacin.

## 2. Materials and Methods

### 2.1. Clinical Isolates

In this study, 19 XDR clinical isolates of MTB were obtained from the Institute for Pulmonology and Phthisiology, Minsk, Belarus, in relation with THE Research Institute for Epidemiology and Microbiology, Minsk, Belarus.

### 2.2. Culture Conditions

The isolates were cultured on Löwenstein–Jensen medium (Merck, Darmstadt, Germany) containing progressively increasing concentrations of ofloxacin (2, 4, 8, 16, 32 mg/L) in slant agar tubes, and were incubated at 37 °C for 28 days. All experiments were done under a class II biosafety cabinet (BSC; vertical, laminar-flow which blows filtered air over work area; Ultra Violet lamp optional).

### 2.3. Inoculation

Suspensions containing approximately 1.5 × 10^8^ colony forming units (CFU)/mL were prepared from MTB grown on ofloxacin-containing medium and were isolated by drug free media in a pre-sterilized laminar hood for aseptic precautions. Pellets were harvested from the suspensions by centrifugation at 7000 rpm for 5 min.

### 2.4. Transmission Electron Microscopy (TEM)

Samples were prepared for TEM examination as described earlier [12].

Briefly, bacterial cells were fixed by 4% (*v*/*v*) glutaraldehyde and 0.1 M sodium cacodylate buffer (Merck) at pH 7.2. Post fixation was carried out in 1% (*w*/*v*) osmium tetroxide in cacodylate buffer for 2 h at room temperature.

In order to examine whole morphology changes (not intracellular parts), negative staining was performed. For this purpose, the samples were placed on 300 mesh copper grids covered by carbon formvar, and excess solution was removed by blotting with filter paper and stained with 5% (*w*/*v*) uranyl acetate in 70% (*v*/*v*) methanol.

The morphology of isolates was examined by Transmission Electron Microscope (Hitachi H7100, Hitachi, Tokyo, Japan).

## 3. Results

### 3.1. Culture Results

Nineteen XDR clinical isolates of MTB grew successfully on Löwenstein–Jensen medium containing ofloxacin.

**Figure 1:**
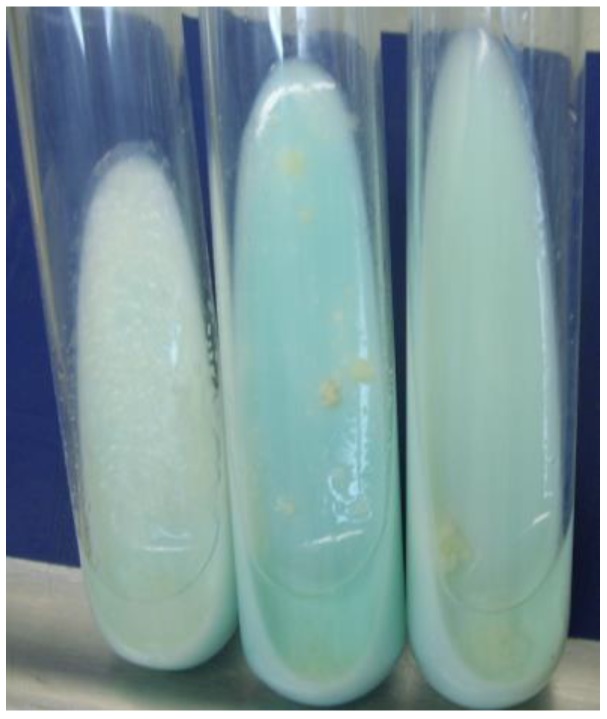
Colony formation of extensively drug resistant tuberculosis (XDR-TB) cells grown on medium with 32 mg/L ofloxacin.

All studied XDR isolates were grown on 2, 4, 8 mg/L, seven isolates on 16 mg/L, and four isolates on 32 mg/L ofloxacin, and grown successfully (Figure 1).

### 3.2. TEM Examinations

**Figure 2:**
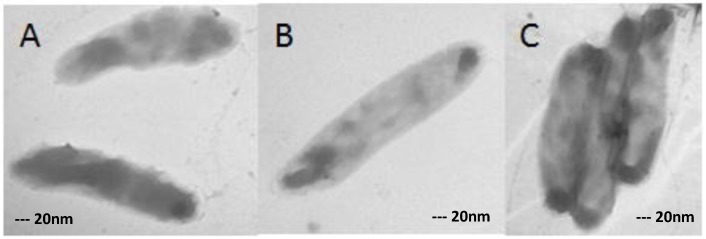
XDR-TB controls (XDR cells grown in medium without any antibiotic) in single (**A**,**B**) and in flock (**C**). Magnification: 40,000×.

**Figure 3:**
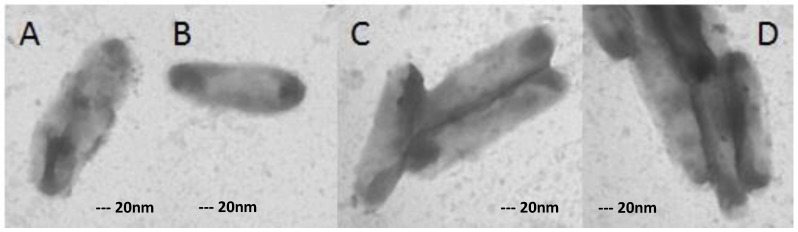
XDR-TB cells grown in medium containing 8 mg/L ofloxacin in single (**A**,**B**) and in flock (**C**,**D**). Magnification: 40,000×.

**Figure 4:**
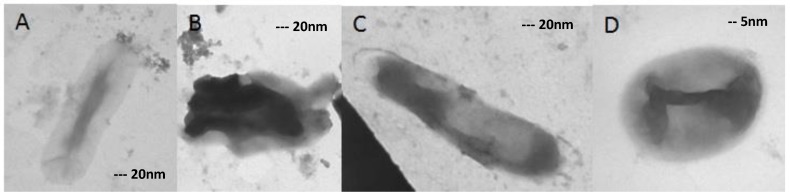
(**A**–**C**) 40,000× magnified and (**D**) 100,000×. The isolates were grown on 16 mg/L ofloxacin, and changes in morphology are clearly distinguishable.

**Figure 5:**
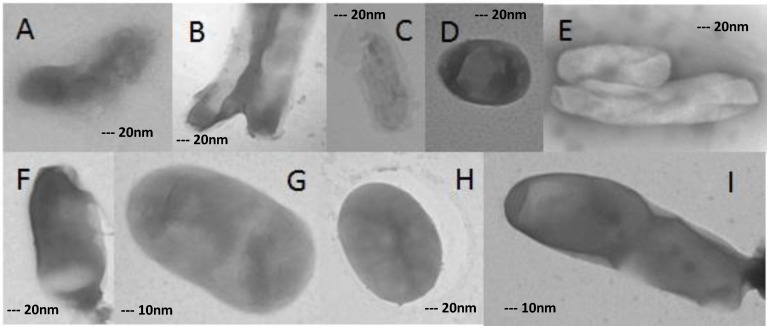
Morphological changes in XDR-TB cells grown on concentration of 32 mg/L ofloxacin. (**A**–**F**,**H**) 40,000×; (**G**,**I**) 80,000×.

TEM images revealed that ofloxacin-resistant XDR MTB isolates in high doses of ofloxacin had marked morphological differences in comparison with isolates grown in medium without any drugs (Figure 2, Figure 3, Figure 4 and Figure 5). In the present study, the TEM images showed marked differences in morphology among the studied isolates. In some XDR cells, an abnormal ultrastructure were shown.

The change of morphology from bacilli form to oval or degraded forms was seen in cultures at the incubation period with antibiotic concentrations.

### 3.3. XDR MTB Grown on Drug-Free Medium

XDR-TB clinical isolates grown in medium without any antibiotics were used as control. As shown in Figure 2, the XDR-TB isolates had normal morphology in single (B) and in flock (C).

### 3.4. XDR MTB Grown on 8 mg/L Ofloxacin

The morphology of bacteria in 8 mg/L ofloxacin had no visible changes, but in Figure 3A, it can be seen that the morphology began to change. Overall, around 10% of the cells had minor changes in morphology. As shown, cells arranged in single form or created small flock (Figure 3).

### 3.5. XDR MTB Grown on 16 mg/L Ofloxacin

Morphological changes were obviously seen in the cells grown on medium containing 16 mg/L ofloxacin.

As shown in Figure 4, changed cells were detected in 55% of all cells. Swollen form (protoplast like) (D), ghost-like cell (A), and degraded forms (B) could be seen in the figure. In a few cases, the cytoplasm was detached from the cell wall (C). 

### 3.6. XDR MTB Grown on 32 mg/L Ofloxacin

At a concentration of 32 mg/L ofloxacin, more than 80% of XDR-MTB cells had abnormal morphologies, as described for the 16 mg/L concentration (Figure 4). However, even in this form, growing cells were detected (Figure 5) (Table 1).

All of them have formed normal colonies in Löwenstein–Jensen medium (Figure 5).

The morphology of MTB cells grown on high doses of ofloxacin was changed but not destroyed. The growth of bacteria in high doses of ofloxacin disagrees with the probability of resistance break by administration of high doses of ofloxacin.

## 4. Discussion

Drug resistance in MTB is still the main reason for the worldwide distribution of tuberculosis disease. Growth and colony forming of MTB on medium containing 2 mg/L ofloxacin means that the bacterium is resistant to ofloxacin. In this study, a few resistant isolates were grown on medium containing ofloxacin with 16 magnitude concentrations (32 mg/mL). It was concluded that we cannot control the drug-resistant strains and treat resistant tuberculosis patients with high dose administration of the drug [2,3], and such high concentrations of ofloxacin would likely be toxic to humans.

Fluoroquinolones such as, norfloxacin, ofloxacin, lomefloxacin, and sparfloxacin have been found to be potent with broad spectra of activity against mycobacteria. Many of these compounds (particularly ofloxacin) show very high activity against *Mycobacterium tuberculosis*, and could penetrate the mammalian cells and act intracellularly [7,8]. Ofloxacin is considered to be a second-generation fluoroquinolone, and is an important drug in tuberculosis cure [14].

Sieniawska et al. found that the essential oil of *Monardella purpurea* had a significant antimycobacterial activity. They proved significant changes in the overall *Mycobacterium* cell shape and cytoplasm uniformity and consistency by TEM evaluation [15].

The ultrastructure of MTB cells undergoing division was examined by electron microscopy in the work by Dahl. He reported two features of cell division and observed cells undergoing a type of snapping post fission movement [16].

In the other work, he showed surface bleb-like structures that accumulate as cultures age. He reported the unusual feature of aging MTB cultures that develop extracellular fibrils which had roles of adhering cells to surfaces [17].

In the present study, we found morphological changes in MTB cells when exposed to high doses of ofloxacin.

Velayati and Farnia reported marked differences in the thickness of resistant and susceptible MTB cell walls as 20.2 and 17.1 nm for the XDR and MDR TB bacilli, respectively, and 15.6 nm for the susceptible isolates by TEM [18].

Hosseini et al. reported that transmission electron microscopy examination revealed two distinct types of pili in MTB isolates. They found a bundle-forming pilus and rope-like ones in 10% of all studied isolates [12].

In this study, the action of ofloxacin on the growth and morphology of *Mycobacterium* cells during exposure to various doses (control, 2, 4, 8, 16 and 32 mg/L) of ofloxacin was investigated. It was shown morphological changes were increased with increasing drug concentration.

## 5. Conclusions

Transmission electron microscopy showed obvious differences among XDR isolates that were not exposed to the drug and the isolates that were exposed to 16 and 32 mg/L concentrations of ofloxacin. It was concluded that several magnitudes of drug doses could not prevent the growth of drug-resistant forms.

## Figures and Tables

**Table 1:** Morphology of *Mycobacterium tuberculosis* (MTB) cells grown on increasing doses of ofloxacin.

Dose (mg/mL)	Destruction	Spherical	Oval	Without Changes
8	0%	0%	43%	57%
16	29.40%	29.40%	17.60%	23.50%
32	32.20%	20.80%	28%	19%

## References

[1] Zager E.M., Mcnerney R. (2008). Multidrug-resistant tuberculosis. BMC Infect. Dis..

[2] World Health Organization (WHO) (2008). Global Tuberculosis Control—Surveillance, Planning, Financing. http://www.who.int/tb/publications/global_report/2008/en/index.html.

[3] World Health Organization (2012). Global Tuberculosis Report 2012. http://apps.who.int/iris/bitstream/10665/75938/1/9789241564502_eng.pdf.

[4] Surkova L., Horevich H.L., Titov L.P., Sahalchyk E., Arjomandzadegan M., Alinejad S., Sadrnia M. (2012). A study on demographic characteristics of drug resistant *Mycobacterium tuberculosis* isolates in Belarus. Int. J. Mycobacteriol..

[5] Ojha A.K., Hatfull G.F., Cardona P. (2012). Biofilms of *Mycobacterium tuberculosis*: New perspectives of an old pathogen. Understanding Tuberculosis—Deciphering the Secret Life of the Bacilli.

[6] Espinal M.A. (2003). The global situation of MDR-TB. Tuberculosis.

[7] Spigelman M., Ma Z. (2004). *Mycobacterium tuberculosis*: New tricks for an old bug. Expert Rev. Anti-Infect. Ther..

[8] Arjomandzadegan M., Titov L.P., Surkova L.K., Farnia P., Sheikholeslami F., Owlia P., Eshghinejad A., Farazi A.A. (2012). Determination of principal genotypic groups among susceptible, MDR and XDR clinical isolates of *Mycobacterium tuberculosis* in Belarus and Iran. Tuberk Toraks.

[9] Arjomandzadegan M., Nazari R., Zolfaghari M.R., Taherahmadi M., Sadrnia M., Titov L.P., Ahmadi A., Shojapoor M. (2015). Performance Assessment of the Polymerase Chain Reaction-Restriction Fragment Length Polymorphism Method for Rapid Detection of Susceptibility to Ethambutol and Molecular Prediction of Extensively Drug-resistant Tuberculosis in Clinical Isolates of *Mycobacterium tuberculosis*. West Indian Med. J..

[10] Hall C.E., Keegan H., Rogstad K.E. (2003). Psychiatric side effects of ofloxacin used in the treatment of pelvic inflammatory disease. Int. J. STD AIDS.

[11] Khrustalev V.V., Arjomandzadegan M., Barkovsky E.V., Titov L.P. (2012). Low rates of synonymous mutations in sequences of *Mycobacterium tuberculosis* GyrA and KatG genes. Tuberculosis.

[12] Hosseini H., Fooladi A.A.I., Arjomandzadegan M., Emami N., Bornasi H. (2014). Genetics study and transmission electron microscopy of pili in susceptible and resistant clinical isolates of *Mycobacterium tuberculosis*. Asian Pac. J. Trop. Med..

[13] Drlica K., Zhao X. (1997). DNA gyrase, topoisomerase IV, and the 4-quinolones. Microbiol. Mol. Biol. Rev..

[14] Nelson J.M., Chiller T.M., Powers J.H., Angulo F.J. (2007). Fluoroquinolone-resistant *Campylobacter* species and the withdrawal of fluoroquinolones from use in poultry: A public health success story. Clin. Infect. Dis..

[15] Sieniawska E., Swatko-Ossor M., Sawicki R., Ginalska G. (2015). Morphological Changes in the Overall *Mycobacterium tuberculosis* H37Ra Cell Shape and Cytoplasm Homogeneity due to *Mutellina purpurea* L. Essential Oil and Its Main Constituents. Med. Princ. Pract..

[16] Dahl J.L. (2004). Electron microscopy analysis of *Mycobacterium tuberculosis* cell division. FEMS Microbiol. Lett..

[17] Dahl J.L. (2005). Scanning electron microscopy analysis of aged *Mycobacterium tuberculosis* cells. Can. J. Microbiol..

[18] Velayati A.A., Farnia P. (2009). Differences in cell wall thickness between resistant and nonresistant strains of *Mycobacterium tuberculosis*: Using transmission electron microscopy. Chemotherapy.

